# Editorial: Transport of Nutrients, Metabolites and Ions Linked to Bioenergetics: Relevance to Human Pathology

**DOI:** 10.3389/fmolb.2021.770797

**Published:** 2021-09-27

**Authors:** Mariafrancesca Scalise, Piotr Koprowski, Cesare Indiveri

**Affiliations:** ^1^ Department DiBEST (Biologia, Ecologia, Scienze della Terra) Unit of Biochemistry and Molecular Biotechnology, University of Calabria, Rende, Italy; ^2^ Nencki Institute of Experimental Biology (PAS), Warsaw, Poland

**Keywords:** transporter, pathology, metabolism, nutrients, bioenergetics

The Research Topic on “*Transport of Nutrients, Metabolites and Ions linked to Bioenergetics: Relevance to Human Pathology*” volume 1, includes 15 contributions among which are 13 original articles and two reviews. This Research Topic aimed to shed further lights on the role of membrane transporters in bioenergetics, a forefront area in both basic and applied research. The number of genes coding for transport proteins increased across evolution from prokaryotes to eukaryotes: several hundreds of membrane transporters are now considered crucial in ensuring cell homeostasis and, hence, life. Indeed, these proteins allow for the flux of molecules that cannot freely cross biological membranes such as nutrients, ions, metabolites, and, last but not least, several xenobiotics including drugs (Nakanishi and Tamai, 2011; Rask-Andersen et al., 2013). In line with this fundamental role, the number of pathologies, which are recognized to be linked with inherited or acquired defects of function/expression/regulation of transporters is continuously increasing (Cesar-Razquin et al., 2015). Since molecular identification of membrane transporters is a relatively novel field of investigation, several gaps still exist in their knowledge. Accordingly, many genes encoding membrane transporters are still classified as orphans, i.e., the gene/protein is known but the functional characterization is not available yet. Some others, which display a longer scientific history, are at an advanced stage of investigation, including the resolution of 3D structures. However, most of the transporters are at an intermediate state of knowledge, thus needing further efforts to complete their characterization (Saier et al., 2021).

The original articles included in the Research Topic describe studies performed with various experimental models and updated technical approaches revealing novel aspects of membrane transporter biology and link with human pathologies.

Regarding the characterization of unknown transporters, Fredriksson’s group performed a study employing a *Drosophila melanogaster* model together with human cell lines revealing that the protein UNC93A is responsible for the regulation of potassium flow through the membrane of renal cells triggering edema development (Ceder et al.).

Regarding membrane proteins whose knowledge is at a more advanced stage, six articles have been included in the Research Topic. In the first one, novel roles for vitamin K (VK) acting on membrane features and bioenergetics have been proposed by Nesci’s group: the article pointed out that the diverse structures of VK vitamers can differently modulate gut functions, via mitochondrial OXPHOS, ranging from osmotic balance to nitrogen recycling (Bernardini et al.). In the next article, Ciarimboli’s group described the expression and function of the Organic Cation Transporter 2 (OCT2) in the pancreas. This protein is a well-known transporter involved in the uptake of several drugs currently employed in the therapy of human pathologies including diabetes and cancers. The presence of OCT2 in the pancreas along with the glucose transporter GLUT2, indicates a functional relationship of the two transporters under high glucose, suggesting that a mutual regulation of the two proteins may affect also the drug disposition in pancreatic cells (Schorn et al.).

The Xu’s group achieved an important milestone in the study of energy homeostasis in thermogenic adipocytes; the article reveals an intriguing role for transferrin receptor Trf1 in monitoring the activation or inhibition of these cells upon external stimuli. The receptor can be considered a novel marker of functional adipocytes in terms of thermogenic program activation and mitochondrial integrity (Qiu et al.). Geyer’s group described the actual substrate specificity of three transporters belonging to the SLC10 family, namely NTCP (SLC10A1), ASBT (SLC10A2), and SOAT(SLC10A6). These proteins are known as hepatic bile acid and steroid transporters, as well as hepatic virus receptors. By employing human cell lines stably overexpressing the three proteins the authors showed that some features, in terms of substrate specificity and inhibition pattern, among the three proteins are overlapping even though differences in active binding sites have been reported. As an example, Troglitazone, BSP, and erythrosine B were revealed to be common inhibitors, while cyclosporine A, irbesartan, ginkgolic acid 17:1, and betulinic acid only inhibited NTCP and SOAT, but not ASBT (Grosser et al.). Another article by Fredriksson’s group suggested a new role for the recently de-orphanized SLC38A10 transporter, an ER/Golgi protein involved in the glutamate/GABA/glutamine cycle. In this article, a role for SLC38A10 in cortical astrocytes in sensing glutamate neurotoxicity and oxidative stress has been proposed, which is linked to the p53 action mechanism (Tripathi et al.). The Indiveri’s group highlighted an unexpected function of the amino acid transporter ASCT2. This protein has been functionally and structurally characterized over the years and its function as neutral amino acid transporter is well assessed. In this article, using the proteoliposome model, the authors showed that ASCT2 mediates the transport of glutamate, as well. The transport of the acidic amino acid occurs by a more complex mechanism involving co-transport of proton besides Na^+^, in exchange with glutamine. These data underlie an additional role for ASCT2 in those tissues in which a glutamate/glutamine cycle occurs. The article also introduced a spectroscopic methodology for measuring transport, based on the use of a fluorescent probe entrapped into the proteoliposomes (Scalise et al.).

An important challenge in the study and characterization of membrane transporters is the use of adequate experimental techniques (Cesar-Razquin et al., 2015). It is not trivial to think that the relatively slow advancement in the knowledge of membrane transporters is mainly due to technical limitations of the classical biochemistry approaches employed in the studies of these highly hydrophobic proteins. Therefore, the research of the most suitable model/method is still a hot spot in this field.

In line with this, the Research Topic hosts three articles dealing with technical advancements. The article by Broer’s group described a GC-MS/Single-Cell method settled up for deciphering substrate specificities and signalling of amino acid transporters, whose redundancy and promiscuity is well recognized. Interestingly, the developed technique is based on the expression of every single transporter in *Xenopus laevis* oocytes, followed by GC-MS analysis of the whole content of single cells after the transport phenomenon. This approach revealed importance in updating the knowledge on some amino acid transporters: indeed, overlapping specificities but also some discrepancies with the published literature data have been described for the analysed proteins. As an example, in contrast to previous data, no substantial accumulation of cationic amino acids seems to be linked to ATB^0,+^ (SLC6A14) and SNAT4 (SLC38A4) expression. In addition, SNAT2 (SLC38A2) was revealed to be a good leucine accumulator (Fairweather et al.). In the same frame, the article by Fotiadis’s group deals with a yeast cell-based assay to search for substrates and inhibitors of two heterodimeric transporters, namely 4F2hc-LAT1 (SLC7A5) and 4F2hc-LAT2 (SLC7A8). These proteins are of great interest in the pharma field due to their involvement in human diseases, including cancers. The technique has been optimized using [^3^H]-L-leucine as the main substrate, and triiodothyronine (T3) and thyroxine (T4) as well BCH and JPH203 (KYT-0353) as inhibitors. This validation opens novel perspectives also for other heterodimeric transporters of interest (Kantipudi and Fotiadis). Finally, Oreb’s group set up a screening system for drugs targeting GLUT based on overexpression of functional GLUT2 and GLUT3 in yeast. This whole-cell system employs yeasts with defects in the glucose uptake, which are rescued upon human transporter expression. The simplicity of handling yeast makes this system a very powerful resource for screening anti-diabetic drugs candidates (Schmidl et al.).

The last corpus of three articles included in the Research Topic is mainly focused on membrane proteins linked to human diseases. In particular, the Bisaccias’s group investigated the effects of citrate in hepatic tumour cells. Very interestingly, the treatment with low or high concentrations of citrate triggered opposite effects on cancer cell proliferation: at low doses, citrate has been reported as a promoter of cancer cell growth, while at high doses, citrate behaved as a Trojan horse for oxaloacetate inside cells with suppression of cell metabolism and consequently inhibition of cancer cell growth (Petillo et al.). Furthermore, a second paper by Geyer’s group analysed the double function of the Na^+^-Taurocholate Co-transporting Polypeptide (NCPT) as both bile acid transporter and hepatitis B/D virus receptor. The authors employed a combined approach of mutational analysis, membrane-based yeast-two hybrid system, and co-immunoprecipitation to unveil structural/functional properties of the NCPT protein overexpressed in hepatic human cell lines. In particular, the authors identified the GXXXG/A motifs in TMD2 and TMD7 as important for proper folding and sorting of NTCP with indirect effects on glycosylation, homodimerization, and bile acid transport of NTCP, as well as its HBV/HDV receptor function (Palatini et al.). Finally, the article of Gu’s group proposed the molecular mechanism underlying Cystic Fibrosis (CF)-related diabetes. The authors demonstrated that this comorbidity in CF patients is due to a short-circuit of the communication between CFTR and the second messenger PI(4,5)P2, responsible for GLUT4 translocation at the plasma membrane (Gu et al.).

The Research Topic includes also two reviews dealing with transporters involved in bacterial nutrient disposition and with detoxification by iron overload. The first one, by Dobson’s group, is a systematic review on membrane transporters responsible for distributing a large variety of nutrients to pathogenic bacteria. This deep examination, in terms of protein-protein interactions and regulation by membrane lipids, has significant outlooks in the identification of novel antimicrobial targets (Davies et al.). The second review, by Sun’s group deals with a high-risk factor, common to different human diseases, i.e., iron overload. The most common alteration of iron metabolism is iron deficiency and then, less attention is paid to iron overload that is detrimental for the body’s homeostasis due to macromolecule peroxidation exerted by iron ions. The review focuses on the chelating properties of several plant-derived flavonoids and their functions as a complementary therapy to treat iron overload. This analysis draws conclusion relevant to studies of flavonoid-delivering molecules to overcome some intrinsic difficulties linked to flavonoid absorption, such as their low water solubility (Wang et al.).

## Concluding Remarks

This Research Topic added important missing pieces to the very large puzzle of the membrane transporter field. However, a systematic description of membrane transporters and channels and their actual role in cell homeostasis is far from being completed and is a long-term objective still requiring consistent efforts both in terms of experimentation, collection, and data analysis, due to the sizable number of players and the heterogeneity of their state of knowledge.
